# Microstructure Evolution and the Resulted Influence on Localized Corrosion in Al-Zn-Mg-Cu Alloy during Non-Isothermal Ageing

**DOI:** 10.3390/ma11050720

**Published:** 2018-05-03

**Authors:** Jun-Zhou Chen, Guo-Ai Li, Xin Cai, Jian-Tang Jiang, Wen-Zhu Shao, Li Yang, Liang Zhen

**Affiliations:** 1 Beijing Institute of Aeronautical Materials, Beijing 100095, China; liguoai_1@163.com; 2 Beijing Engineering Research Center of Advanced Aluminum Alloys and Application, Beijing 100095, China; 3 School of Materials Science and Engineering, Harbin Institute of Technology, Harbin 150001, China; Caixin.0910@163.com (X.C.); jjtcy@hit.edu.cn (J.-T.J.); wzshao@hit.edu.cn (W.-Z.S.); hit-yl@163.com (L.Y.); 4 National Key Laboratory of Precision Hot Processing of Metals, Harbin Institute of Technology, Harbin 150001, China

**Keywords:** Al-Zn-Mg-Cu alloy, non-isothermal ageing, precipitation, segregation, local corrosion

## Abstract

A non-isothermal ageing process was proposed for an Al-Zn-Mg-Cu alloy aiming to accommodate the slow heating/cooling procedure during the ageing of large components. The evolution of microstructure and microchemistry was analyzed by using transmission electron microscopy, high-angle annular dark field imaging, and energy dispersive spectrometry. The age-hardening of the alloy was examined to evaluate the strengthening behavior during the non-isothermal process. The corrosion behavior was investigated via observing the specimens immersed in EXCO solution (solution for Exfoliation Corrosion Susceptibility test in 2xxx and 7xxx series aluminum alloys, referring ASTM G34-01). Secondary precipitation was observed during the cooling stage, leading to increased precipitate number density. The distribution of grain boundary precipitates transits from discontinuous to continuous at the cooling stage, due to the secondary precipitation’s linking-up effect. The solutes’ enrichment on grain boundary precipitates and the depletion in precipitate-free zones develops during the heating procedure, but remains invariable during the cooling procedure. The corrosion in NIA (Non-isothermal Ageing) treated specimens initiates from pitting and then transits to intergranular corrosion and exfoliation corrosion. The transition from pitting to intergranular corrosion is very slow for specimens heated to 190 °C, but accelerates slightly as the cooling procedure proceeds. The transition to exfoliation corrosion is observed to be quite slow in all specimens in non-isothermal aged to over-aged condition, suggesting a corrosion resistance comparable to that of RRA condition.

## 1. Introduction

The Al-Zn-Mg-Cu series of aluminum alloys have been widely used for fabrication of bearing components, including wing spars, stringers, bulkheads, and so on, in the aircraft industry, for the past a few decades [1,2,3,4,5,6,7]. High strength, high toughness, high damage tolerance, as well as satisfactory corrosion resistance, is usually required, simultaneously, in the designing of modern aircrafts, for the consideration of high structure efficiency, high reliability, high safety, and long service life [1,2,3,4,5,6,7]. Al-Zn-Mg-Cu alloys, however, exhibit high susceptibility to localized corrosion (LC), especially for under- and peak-aged conditions [8,9,10,11,12], which partially restricts their application. 

LC usually initiates from pitting, gradually evolves to crevice, intergranular corrosion (IGC), or exfoliation corrosion (EFC), and eventually propagates deep into the structure [12,13]. Various types of LC, especially pitting and IGC, can serve as potential sites for crack initiation, and thus, induce stress corrosion cracking (SCC) or corrosion fatigue failure [14,15,16]. Thus, reducing the susceptibility to LC is an important mission for the development and application of Al-Zn-Mg-Cu alloys. 

Many efforts have been made to decrease Al-Zn-Mg-Cu alloys’ susceptibility to LC. Besides optimizing the composition [17,18,19], different ageing processes have been established to balance mechanical performances and LC resistance to meet comprehensive requirements from aircraft fabrication [20,21,22,23,24]. Two-staged T7x ageing was proven to be effective in decreasing the susceptibility to LC, compared with T6 ageing, at a cost of 10–30% strength reduction [20,21,22,23,24,25]. The development of T77 ageing has presented an effective way for obtaining satisfactory LC resistance without compromising mechanical performance [23,24,25], which has then greatly promoted the application of 7150 and 7055 Al alloys [1,2,3,4]. T77 ageing, however, can hardly be applied on large components with thick sections, since the transitory retrogress procedure is difficult to carry out [26,27,28,29,30]. A new challenge for ageing processes has then emerged, as the need for ageing large components increases very quickly [1,2,7]. To solve the problem, non-isothermal ageing (NIA) was developed to accommodate the long-lasting heating and/or cooling procedure during the ageing of large components. The merits [31,32,33,34,35] of non-isothermal ageing have been observed as excellent performance and/or improved efficiency. These research studies suggested the possibility to integrate non-isothermal procedures into ageing, but the feasibility needs to be examined, in view of microstructure evolution, as well as performance variations.

On the other hand, the correlation between the microstructures and LC remains unclear in Al-Zn-Mg-Cu alloys. The initiating and the developing of LC is believed related to the microstructure that established during the ageing. Specifically, features of grain boundaries (GBs) and adjacent regions, including the grain boundary precipitates (GBPs), the precipitate free zones (PFZs), and the adjacent matrix, are believed dominate the LC process in Al-Zn-Mg-Cu alloys [9,36,37]. The potential difference between precipitates and the matrix is likely to initiate the pitting, due to the galvanic effect or the preferential dissolution. The galvanic effect may also occur on grain boundaries due to the potential deficiency between PFZs and adjacent regions [38], which is also related to the local chemistry diversity that establishes during the ageing [39,40,41,42]. Besides the composition, GBPs’ distribution may also affect the LC. Continuously-distributed GBPs were believed to present an active path for the propagating of localized corrosion [9,29]. Although many studies have been carried out, morphologic observation and analysis of the LC process can be informative for understanding LC’s initiation and development.

The current work was initiated to investigate the precipitation during the NIA process, and to address the influence on localized corrosion. Comparison in microstructure and the corrosion process with that in the T6 and RRA (Retrogression Re-Aging) condition was also systematically carried out to evaluate the particularity of the NIA treating.

## 2. Experimental

**Figure 1:**
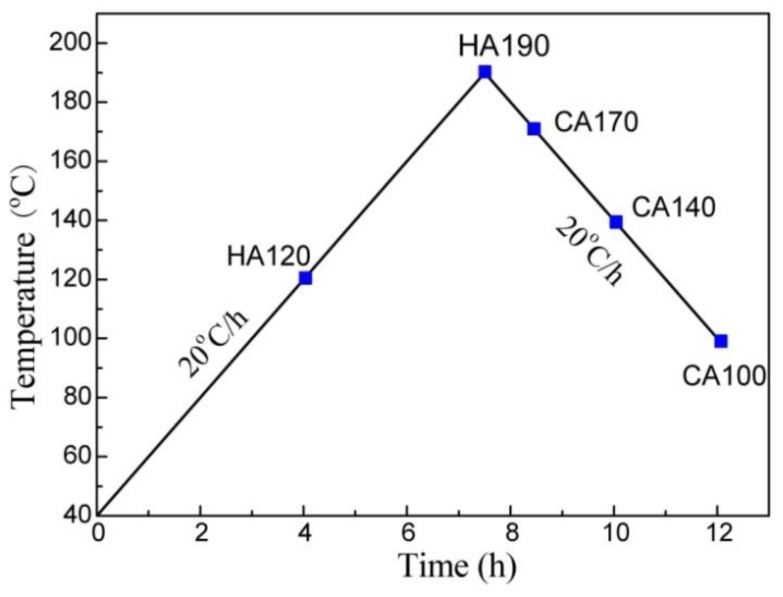
Non-isothermal ageing procedure utilized for the Al-Zn-Mg-Cu alloy. The alloy was first heated to 190 °C, and then allowed to cool to 100 °C. Both of the ramping and cooling rates are set at 20 °C/h. Squares denote the samples for microstructure observation.

A hot-rolled and tempered Al-6.0Zn-2.3Mg-2.0Cu (wt %) plate was used in this work. The specimens were solution treated in salt bath at 477 °C for 1 h, and quenched into cold water, prior to non-isothermal ageing. For non-isothermal ageing treatment, specimens were heated from 40 °C at a ramping rate of 20 °C/h to 190 °C, and then cooled down to a certain ending temperature at a cooling rate of 20 °C/h, and then quenched into cold water, as demonstrated in the diagram (see Figure 1). The specimens heated from 40 °C to 120 °C and 190 °C were named HA120 and HA190, respectively. The specimens heated to 190 °C and then cooled to 170 °C, 140 °C, and 100 °C, and quenched, were named CA170, CA140, and CA100, respectively. RRA treatment (120 °C × 24 h + 200 °C × 0.75 h + 120 °C × 24 h) and T6 ageing (120 °C × 24 h) were performed in parallel for comparison.

The microstructures of NIA-treated specimens were observed on a transmission electron microscope (TEM, Tecnai G^2^ F30, FEI, Hillsboro, OR, USA) operated at 300 kV to analyze the precipitation behavior. Atomic number contrast (Z-contrast) tomography was taken to analyze the local compositions by performing HADDF-STEM observing across GBs. Energy-dispersive X-ray spectroscopy (EDS, EDAX Inc., Hoffman Estates, IL, USA) line-scanning was carried out to quantitatively characterize the solute concentration profile across selected GBs in STEM mode. The step for line-scanning is 1 nm. The counter of each alloying element to that of Al was recorded sequentially along the selected path. The data collected from the line-scan were normalized to get the intensity of each element. The variation in the intensity can characterize the solute content semi-quantitatively.

Vickers hardness of the NIA specimens was tested to investigate the age-hardening behavior. A load of 0.2 kgf and a hold duration of 40 s were applied. Hardness of specimens of RRA and T6 condition was also measured. The electrical conductivity was measured on a micro-Ohm meter using four probe method. Needle-like specimens, 0.7 × 0.7 mm^2^ in section and 40 mm in length, were used for the conductivity measuring. The corrosion process along an EXCO solution immersing of selected specimens (HA120, HA190, CA100, RRA, and T6) was investigated, referring to the procedure in ASTM G34 [43]. Selected samples were immersed in EXCO solution for 5, 10, and 15 h, and flushed thoroughly before being etched in nitric acid (30%). The etched specimens were observed on scanning electron microscope (SEM, FEI Quanta 200F, FEI, Hillsboro, OR, USA) to examine the surface morphology. Specifically, the pitting, crevice, IGC, EFC, and the transition between them were examined, to figure out the corrosion process. 

## 3. Results and Discussion

### 3.1. Precipitation on the Matrix

**Figure 2:**
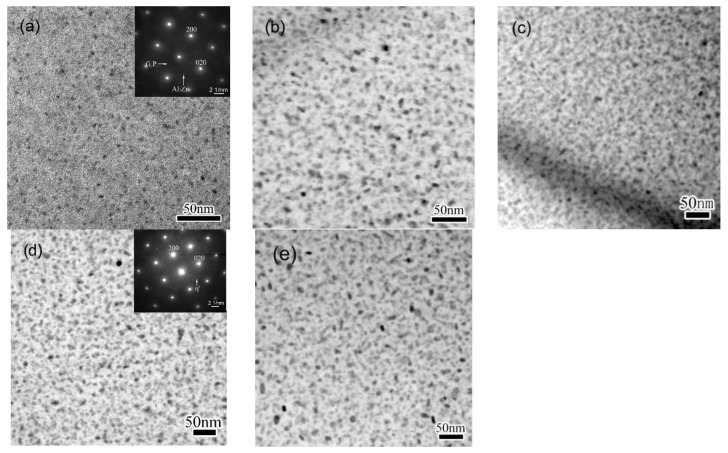
Typical bright-field TEM images of the matrix precipitates in an Al-Zn-Mg-Cu alloy under different non-isothermal ageing conditions, (**a**) HA120; (**b**) HA190; (**c**) CA170; (**d**) CA140; (**e**) CA100. All the images were taken near [001]_Al_. Insets in (**a**,**d**) are SAED patterns showing the formation of GP zone and *η’* phase at different stages.

The size and distribution of matrix precipitates of specimens subjected to various NIA processes were examined by TEM, and results were shown in Figure 2. Selected-area electron diffraction (SAED) patterns of the precipitates were shown as insets of Figure 2a,d. High density of precipitates with very fine sizes can be observed in specimen HA120, as shown in Figure 2a. SAED pattern reveals that matrix precipitates of this condition are mainly GP zones. As for the specimen heated to 190 °C, precipitates are observed grown up to 5–12 nm, and the number density decreases evidently, compared to that of HA120, as shown in Figure 2b. After the specimen is cooled from the peak temperature down to 170 °C, previously existing precipitates are found to coarsen slightly, as shown in Figure 2c. Meanwhile, the number density of precipitates increases greatly, and the image becomes fussy. The fuzziness observed in the TEM image is believed to be induced by the coherent strain, suggesting the presence of very fine precipitates. A similar behavior was previously observed in an Al-Zn-Mg-Cu alloy of NIA condition, and the fine precipitates were identified as secondary precipitates [34,35]. When the specimen is further cooled down to 140 °C, the fussiness that was observed in CA170 condition weakens off, as shown in Figure 2d, indicating the vanishing of the coherent mismatch. The SAED inset in Figure 2d suggests that precipitates of this condition are mainly metastable *η′* phases of MgZn_2_. When further cooled to 100 °C, the precipitates do not change apparently in shape, size, or distribution, compared to CA140, as shown in Figure 2e.

**Figure 3:**
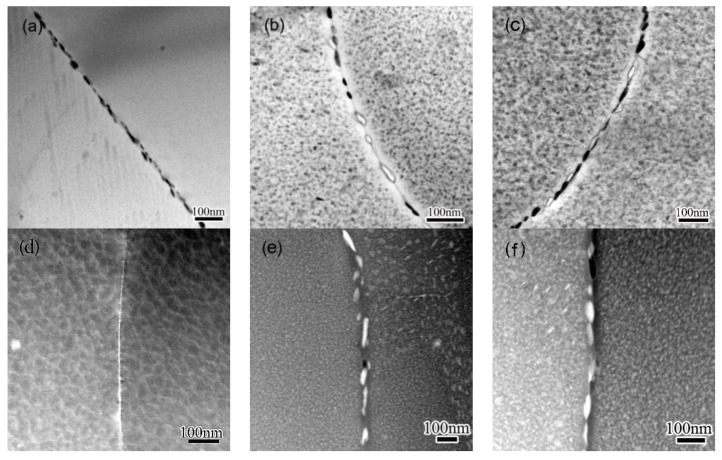
TEM images and HADDF-STEM images showing precipitates and the PFZ along grain boundaries in specimens NIA treated to different conditions: (**a**,**d**) HA120; (**b**,**e**) HA190; (**c**,**f**) CA100.

The microstructure evolution of GBs and adjacent regions during the NIA process was observed, and the results are shown in Figure 3. To examine the distribution of solutes, HADDF-STEM observation was performed across GBs, and the obtained Z-contrast images are also shown in Figure 3. GBPs around 10 nm in width distribute continuously along GBs when the alloy was heated to 120 °C, as shown in Figure 3a. A light gray line about 10 nm in width can be recognized along the GB in the Z-contrast images (Figure 3c), indicating the solutes’ initial segregation on GBs. PFZ can hardly be distinguished in this condition, either from TEM or from HADDF-STEM observation. GBPs 15–30 nm in thickness and 40–100 nm in diameter are observed in HA190, as shown in Figure 3b. These GBPs distribute along the GB at intervals of 30–60 nm. The color of these GBPs become white in HA190, as shown in the Z-contrast image in Figure 3d, suggesting enhanced concentration of Zn and/or Cu. When specimens were cooled to 100 °C, formerly discontinuously distributed GBPs are found linked to each other by fine precipitates between them, as observed in Figure 3d. A similar variation is observed in the Z-contrast images in Figure 3f, but the contract of GBP are quite near to that observed in specimen HA190.

According to the TEM observation, extra precipitation occurs both within the matrix and on GBs during the cooling procedure. A similar phenomenon was previously observed in a 7050 Al alloy when subjected to a T6I6 treatment [26,27], when low temperature ageing was performed after conventional T6 ageing. The precipitation that occurs during the cooling stage was classified as secondary precipitation, and the precipitation that occurs during the conventional T6 is accordingly named primary precipitation. The secondary precipitation is related to the descending temperature in the current study. The matrix remains slightly supersaturated during the cooling, since the solute’s solubility decreases continuously as the temperature decreases, and the superfluous solutes then continuously separate from the matrix. The supersaturation of the matrix is very low during the cooling ageing, and the secondary precipitation is thus weak, and precipitates are, thus, very fine. Most secondary precipitates coarsen quickly once exposed to high temperatures, as observed in 170–140 °C span, but those forms at low temperature may remain fine, as seen at the 140–100 °C stage. A similar behavior of secondary precipitation was previously observed during a previous study in Al-Zn-Mg-Cu alloy [34,35].

### 3.2. EDS Analysis

**Figure 4:**
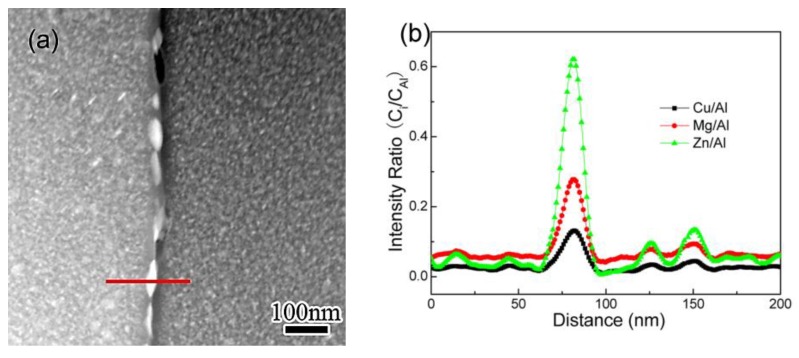
The location of the EDS line-scan, as indicated in red line in (**a**), and the obtained concentration profile of solute (**b**), in CA100.

EDS line-scan was performed to examine the solutes’ concentration profile across grain boundaries. The result obtained in specimen CA100, as a representative one, are shown in Figure 4. The scan was carried out across a selected GBP with a step of 1 nm along the path, marked with a red line in Figure 4a. The curves shown in Figure 4b demonstrate the concentration profiles of solutes obtained from the scan. The intensity ratio (C_i_/*C*_Al_) reveals the relative content of each solute in GBs and PFZs. Zn concentration in GBPs is the most intense, with a highest intensity ratio C_Zn_/C_Al_ of 0.62 being detected. Meanwhile, the depletion of Zn in PFZ is most evident as the lowest intensity ratio (C_Zn_/C_Al_ = 0.02) observed. The C_Zn_/C_Al_ detected within grains is 0.03–0.04, much lower than that in GBPs, but notably higher than that in PFZs. A similar behavior of enrichment and depletion is observed for Mg and Cu, but the intensities vary one from each other.

**Figure 5:**
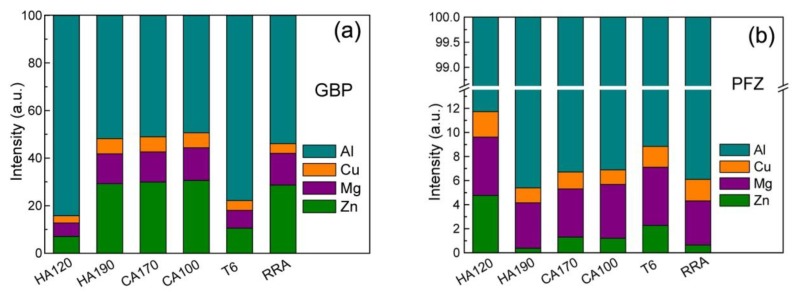
The normalized intensity of each solute calculated from the C_i_/C_Al_ ratios measured in the EDS line-scans: (**a**) the center of GBPs; (**b**) PFZs. Here the intensity of characterized X-ray of each solute was used to evaluate the relative content.

The results of EDS line-scan obtained in specimens of different conditions are listed in the histogram in Figure 5. The segregation of Zn, Mg, and Cu in GBPs is quite weak when the specimen is firstly heated to 120 °C, as shown in Figure 5a. Solutes’ concentration develops quickly as the heating proceeds, and an intensive enrichment is then developed, as a high content is determined for Zn, Mg, and Cu in HA190. The content of Zn and Mg increases persistently, but only slightly, during the subsequent cooling procedure, while the content of Cu remains at around 6.4 at % all through the cooling. The content of Zn, Mg, and Cu observed in T6 condition is a little higher than that in HA120, but still very low. The segregation observed in RRA condition is quite similar to that in specimens obtained at the late stage (190–100 °C span) of NIA process. More specifically, the content of Cu and Zn in specimen HA190, CA170, and CA100 are all slightly higher than those in RRA conditions, but the Mg content is a little lower.

The compositions of PFZs in specimens of different conditions are shown in Figure 5b. Solute depletion in the PFZs is not quite intensive in HA120 condition as the content is still higher than that of the bulk composition. When heated to 190 °C, the depletion of Zn becomes so intensive that Zn can hardly be detected in PFZs in HA190. The Zn content increases during the 190–170 °C span, and then decreases slightly when further cooled to 100 °C. The Mg content in PFZs increases persistently all through the cooling procedure. The Cu content varies slightly as the cooling procedure proceeds, but remains at low levels. The solutes’ depletion is weak in T6 condition, as a high content of solutes is detected, but is slightly stronger than that in HA120. PFZs in RRA condition have a similar content of Zn and Mg, but a higher content of Cu to compared to HA190. On the other hand, PFZs in RRA condition have a lower content of Zn and Mg, but higher content of Cu, compared with CA170 and CA100. It is supposed that, the high temperature that is applied in HA190 and RRA processes contributes to the intensive depletion of Zn in PFZs. To compare with the difference observed in GBPs’ composition, PFZs of different conditions possess similar compositions, which is consistent with the previous analysis [13]. 

### 3.3. The Ageing Hardening 

**Figure 6:**
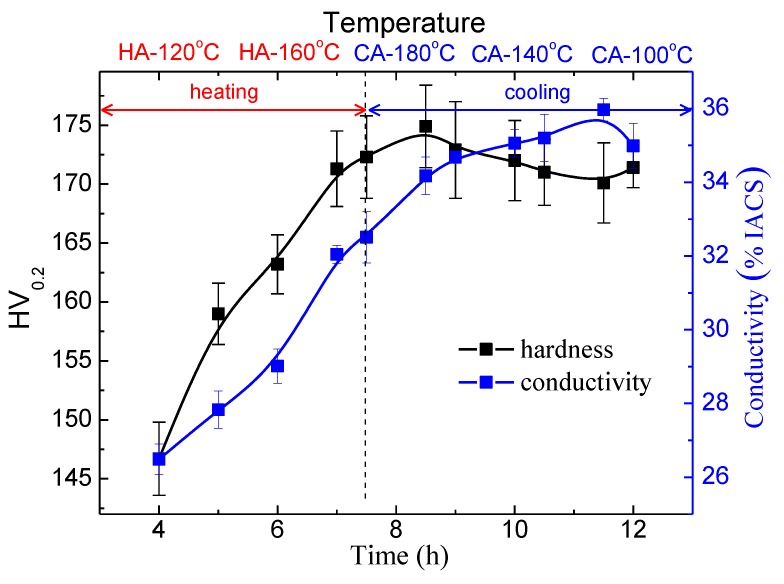
Variation of hardness of the alloy during NIA treatment.

The variation of the hardness during the NIA treatment is demonstrated in Figure 6. The hardness is found to increase all through the heating procedure, and the 190–170 °C stage of the cooling procedure. A peak hardness of HV175 is observed in CA170, which is slightly lower than that of T6 (HV185) and RRA condition (HV179). After the long-lasting increase, the hardness decreases gradually in the 170–120 °C span, and a minimal hardness of 170 HV is observed in CA120. An increase of the hardness is observed once again at the terminal stage, when specimen is further cooled to 100 °C, but is quite slight. 

The continuous increase in precipitates is believed to contribute to the hardening in the heating procedure. Secondary precipitation occurs in the specimens once they are subject to the cooling process, which then results in an increased precipitate number density, and thus, contributes to the hardening of this stage [26,27]. Since precipitates formed from the secondary precipitation are quite fine in size, they coarsen quickly once formed, and their strengthening effect thus decreases [26,27]. When the temperature decreases further to below 110 °C, the secondary precipitation continues to occur, but the formed precipitates do not coarsen, apparently, since the temperature is too low to drive an evident coarsening. The fine precipitates present additional strengthening at the terminal stage, although the contribution is limited. 

The conductivity increases almost all through the NIA process, but exhibits a slight decrease at the terminal stage (110–100 °C). The precipitating, as well as the coarsening of the precipitates, either the primary or the secondary ones, contributes to the increase in conductivity. Very fine precipitates form at the terminal stage, just as discussed above, however, may induce intense lattice distortion, and thus, negatively contribute to the conductivity [28]. 

### 3.4. Localized Corrosion Behavior 

#### 3.4.1. Observations of the LC Process

**Figure 7:**
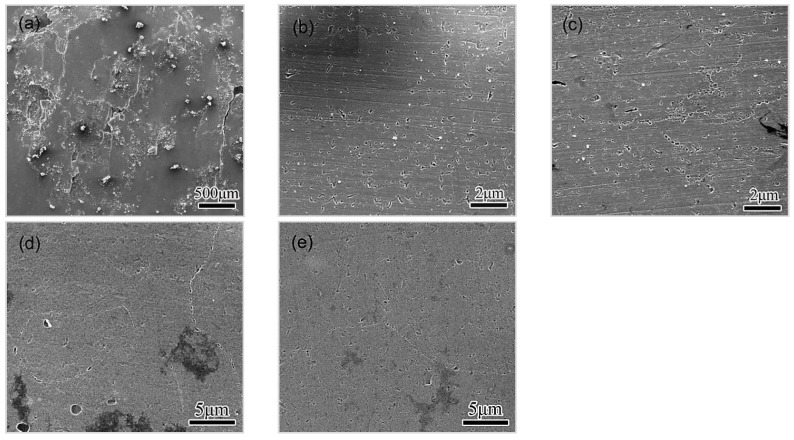
SEM images taken from surfaces exposed to EXCO solution for 5 h, with specimens of different conditions, (**a**) HA120; (**b**) HA190; (**c**) CA100; (**d**) T6 condition; (**e**) RRA condition. Corrosion products were removed before SEM observation.

Specimens of different conditions were immersed in EXCO solution for different durations, flushed thoroughly, and then etched in a nitric acid before SEM observation. Representative images of the corroded surface of specimens immersed for 5 h are shown in Figure 7. Specimen HA120 presents characteristics of peeling along GBs on the corroded surface, and little pitting is observed, as shown in Figure 7a, suggesting the rapid development of IGC in the specimen. As for HA190 condition, many pits are observed, distribute uniformly all through the surface, and the preference of GBs to LC is not obvious, as shown in Figure 7b. A high density of pits is also observed in CA100, either within the matrix or along GBs, as shown in Figure 7c, and a preference for GBs becomes evident. For the T6 and RRA condition, a lot of pits are observed distributed along GBs, but fewer within the matrix, as shown in Figure 7d. Corrosion pits are observed on GBs and within grains, as well as in the specimen of RRA condition, as shown in Figure 7e, suggesting that the preference for GBs is slight. Observation of specimens immersed for 5 h suggests that, specimens of HA120 and T6 condition present higher susceptibility to intergranular corrosion, compared with those in HA190 and RRA condition.

**Figure 8:**
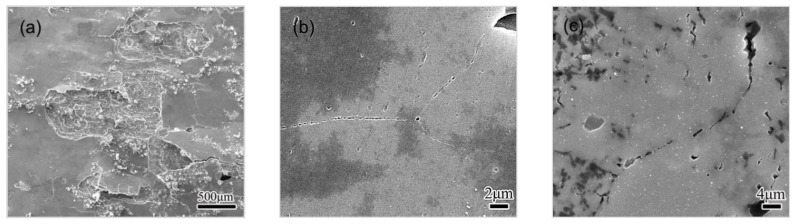

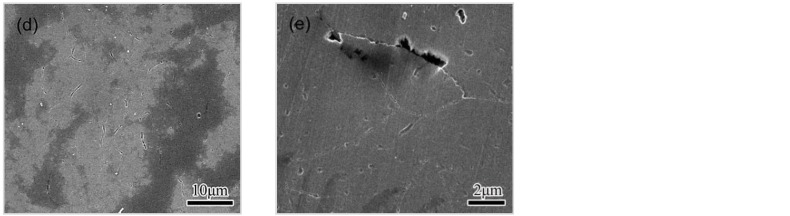
SEM images taken from surfaces exposed to EXCO solution for 10 h, with specimens of different conditions, (**a**) HA120; (**b**) HA190; (**c**) CA100; (**d**) T6 condition; (**e**) RRA condition. Corrosion products were removed.

The spalling in HA120 becomes more severe during the 5–10 h immersion, as shown in Figure 8a, suggesting the quick development of EFC. As for the HA190 condition, pits are found to link up together to form some grooves along GBs, as shown in Figure 8b, suggesting a transition from pitting to IGC. IGC can be observed much more clearly in CA100, as deep and sharp grooves shapes, up along GBs. Pits’ linking-up along GBs are also observed in T6 and RRA condition, as shown in Figure 8d,e. However, grooves observed in the RRA aged specimen are shallower and duller compare with those observed in T6 condition. The pits’ linking-up and the subsequent penetration are supposed to contribute to the transition from pitting to IGC. The pitting–IGC transition develops quickly in CA100 and T6 condition, but solely in HA190 and RRA condition. The comparison between HA190 and CA100 reveals that, the pitting–IGC transition accelerates as the cooling ageing proceeds. 

**Figure 9:**
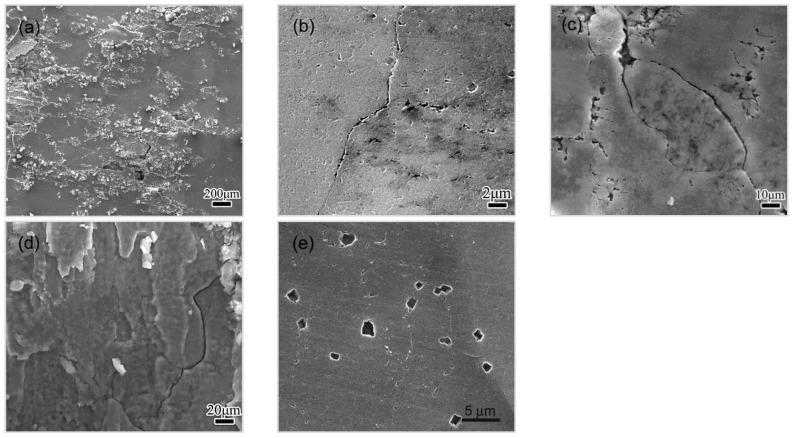
The SEM images taken from surfaces exposed to EXCO solution for 15 h, with specimens of different conditions, (**a**) HA120; (**b**) HA190; (**c**) CA100; (**d**) T6 condition; (**e**) RRA condition. Corrosion products were removed.

The morphology of corroded surfaces exposed to EXCO solution for 15 h are shown in Figure 9. The EFC in HA120 becomes very severe, as shown in Figure 9a. The corrosion has propagated deeply into the materials in HA190, as shown in Figure 9b, indicating the IGC’s development. As for CA100 condition, some GBs (or subgrain boundaries) are observed to be dissolved away, the deep crevices thereafter shape up along GBs, as shown in Figure 9c. The morphology clearly indicates the occurrence of severe IGC. It can thus be inferred that IGC has been developing persistently during the 10~15 h period. No blister or peeling was observed on the corroded surface of CA100 according to the SEM observation, which suggests that the transit to EFC has not occurred. As for T6 condition, the IGC becomes excessive, and the EFC is initiated as cracks were observed, propagated along GBs during the 10–15 h span, as shown in Figure 9d. Some large pits are observed in RRA condition, as shown in Figure 9e, but no sign of IGC or EFC can be observed. It looks like the transition from the pitting to the IGC is much slower in RRA condition than that in HA 190 or CA100 condition.

It can then be concluded that the corrosion behavior, including the initiation and the development, is closely related to the ageing condition. HA120, which is of under-aged condition, exhibits high susceptibility to IGC and EFC. When the alloy is heated to 190 °C, the GBs’ preference for being eroded is greatly weakened, and the transition from the pitting to IGC is also slowed down. The treatment during the cooling stage, however, revives the selective corrosion of GBs, and leads to an accelerated transition from pitting to IGC, as observed in CA100. As a comparison, specimens of T6 condition exhibit a high GB preference in pitting. The selective pitting, together with the subsequent linking-up, causes a rapid development of IGC, and subsequently, of EFC. Specimens of RRA condition also present an obvious GB preference pitting, but the subsequent evolution towards IGC is quite mild. 

According to the comparison, specimens of RRA condition possess the highest resistance to LC, and those of HA120 condition possess the lowest one. The corrosion resistance improves during the heating procedure of the NIA treatment, reaching at a peak level at 190 °C, and then decreasing slightly as the cooling procedure proceeds. A specific comparison shows a similarity in the initiation, pitting–IGC transition, and in the LC process in specimens of HA190 and RRA condition. The similarity in the compositions of GBPs and PFZs contribute to the similar behavior in pitting at the initial stage. The sparse distribution of GBPs in these two conditions contributes to the mild pitting–IGC transition. The similarity confirms the correlation between the microstructure and the corrosion.

#### 3.4.2. Effect of Microstructure and Microchemistry on Intergranular Corrosion

The intergranular corrosion was mainly attributed to the difference in potential between the matrix and GBPs, and is influenced by the microstructure and solute concentration gradients [40]. Though the effects of these parameters are not fully understood, most evidence shows that increasing the size and interspacing, as well as the copper content of the GBPs, decreased the corrosion susceptibility [39,40].

For the NIA-treated specimens, variations in microchemistry and microstructure in GBs and adjacent regions are believed to contribute to the diversity of the corrosion [36,37,38]. GBPs in HA120 possess low concentrations of Cu, and thus, present a potential evidently lower than that of the adjacent matrix and the PFZ [13,22]. A galvanic corrosion thus occurs, leading to the intensive selective corrosion of GPBs. Furthermore, the galvanic corrosion propagates easily along GBs, since GBPs distribute continuously, which facilitate an uninterrupted dissolution of the GB, as noted in previous studies [9,29]. 

The enrichment of Cu in GBPs, as well as the depletion of Zn and Mg in PFZ, becomes evident when the specimen was put into procedures with elevated temperature, which is consistent to that noted in previous studies [41,42]. This variation in local composition leads to decreased potential difference ,and the galvanic effect between GBPs and the matrix weakens, and preferential corrosion of GB can thus be suppressed. Additionally, the sparse distribution of GBPs can interrupt the corrosion’s propagation along GBs, leading to a slower transition from the pitting to IGC [39]. 

The solutes’ enrichment in GBPs, as well as the depletion in PFZ, develops slightly during the cooling procedure. The distribution of GBPs, however, transitions from sparse to continuous, as seen in Figure 3. This variation in the microstructure does not apparently affect the initiation of corrosion, as seen in Figure 7, however, the transition from pitting to IGC can be accelerated (see Figure 7, Figure 8 and Figure 9), which leads to a degraded LC resistance. The continuously distributed GBPs present an active way for the corrosion to propagate [9,29].

Based on the contrastive analysis, it can be concluded that the evolving of local chemistry and GBPs’ distribution separately domains the corrosion process during different stages. The enrichment of solutes, specifically Cu in GBPs, is favorable for improving the resistance to the initiation of LC by degrading the potential difference between the GBPs and their surroundings. This is verified as the corrosion resistance observed in the initial stage goes in the following sequence: HA190 > CA100 > RRA > T6 > HA120. The distribution of GPBs, on the other hand, contributes mainly to the transition from pitting to IGC (EFC). The effect is verified during the cooling stage when the transition accelerates once the GBPs’ continuous distribution emerges. Similar discoveries were previously presented in ref. [40], in which the improvement in IGC resistance observed in RRA, DRRA, and T74 tempered (comparing to that of T6) 7085 Al alloy was attributed not only to the increased size and the interspaces of GBPs, but also the increased Cu content of GBPs. The secondary precipitation, which contributes positively to the mechanical performance when occurring within grains, leads to a degraded corrosion resistance, which occurs on GBs for the linking-up of the existing GBPs.

## 4. Conclusions

The current study focused on the evolution in the microstructure and the microchemistry in an Al-Zn-Mg-Cu alloy during NIA treatment. Some comparisons using RRA and T6 condition as references were also carried out to understand the evolution and the resulting influence on properties.

(1) The precipitation as well as the coarsening develops very quickly when specimens are exposed to an increasing temperature. A secondary precipitation occurs when a cooling procedure is introduced, leading to increased number density of precipitates. The secondary precipitation on GBs contributes to the linking-up of primary GBPs.
(2) A peaked hardness comparable to that of RRA condition is obtained when heated to 190 °C. The secondary precipitation contributes to the increase in hardness at the terminal stage, although a slight coarsening of primary precipitates occurs.
(3) The solutes’ enrichment in GBPs, as well as the depletion in PFZs, develops as the NIA proceeds, and reaches a peak level when the heating procedure ends. The cooling does not influence the enrichment in GBPs evidently, but weakens the depletion of Zn in PFZ.
(4) Enrichment of solutes in GBPs, as well as the depletion in PFZs, is believed decrease the pitting’s preference for GBs. GBPs that continuously distribute along GBs, on the other hand, accelerate the pitting–IGC transition.
(5) The NIA process endues strength and LC resistance comparable to that of RRA condition, and simultaneously, accommodates non-isothermal procedures. It is then a promising method to age large components of aluminum alloys properly.

## References

[1] Starke Jr E.A., Staley J.T. (1996). Application of modern aluminum alloys to aircraft. Prog. Aerosp. Sci..

[2] Staley J.T., Liu J., Warren H. (1997). Aluminum alloys for aerostructures. Adv. Mater. Processes.

[3] Williams J.C., Starke E.A. (2003). Progress in structural materials for aerospace systems. Acta Mater..

[4] Woodward R. (1989). Developments in Aluminum Alloys. Mater. Des..

[5] Liu J., Kulak M. (2000). A new paradigm in the design of aluminum alloys for aerospace applications. Mater. Sci. Forum.

[6] Warner T. (2006). Recently-developed aluminum solutions for aerospace applications. Mater. Sci. Forum.

[7] Heinz A., Haszler A., Keidel C., Moldenhauer S., Benedictus R., Miller W.S. (2000). Recent development in aluminum alloys for aerospace applications. Mater. Sci. Eng. A.

[8] Marlaud T., Malki B., Henon C., Deschamps A., Baroux B. (2011). Relationship between alloy composition, microstructure and exfoliation corrosion in Al-Zn-Mg-Cu alloys. Corros. Sci..

[9] Xiao Y.P., Pan Q.L., Li W.B., Liu X.Y., He Y.B. (2012). Exfoliation corrosion of Al-Zn-Mg-Cu-Zr alloy containing Sc examined by electrochemical impedance spectroscopy. Mater. Corros..

[10] Andreatta F., Terryn H., de Wit J.H.W. (2003). Effect of solution heat treatment on galvanic coupling between intermetallics and matrix in AA7075-T6. Corros. Sci..

[11] Andreatta F., Terryn H., de Wit J.H.W. (2004). Corrosion behaviour of different tempers of AA7075 aluminum alloy. Electrochim. Acta.

[12] Meng Q., Frankel G.S. (2004). Effect of Cu content on corrosion behavior of 7xxx series aluminum alloys. J. Electrochem. Soc..

[13] Ramgopal T., Gouma P.I., Frankel G.S. (2002). Role of grain-boundary precipitates and solute-depleted zone on the intergranular corrosion of aluminum alloy 7150. Corrosion.

[14] Harrison T.J., Crawford B.R., Loader C., Clark G., Brandt M. (2016). Predicting the likely causes of early crack initiation for extruded aircraft components containing intergranular corrosion. Int. J. Fatigue.

[15] Liao M., Renaud G., Bellinger N. (2007). Fatigue modeling for aircraft structures containing natural exfoliation corrosion. Int. J. Fatigue.

[16] Burns J.T., Kim S., Gangloff R.P. (2010). Effect of corrosion severity on fatigue evolution in Al-Zn-Mg-Cu. Corros. Sci..

[17] Deng Y., Yin Z.M., Zhao K., Duan J.Q., Hu J., He Z.B. (2012). Effects of Sc and Zr microalloying additions and aging time at 120 degrees C on the corrosion behaviour of an Al-Zn-Mg alloy. Corros. Sci..

[18] Meng C.Y., Zhang D., Zhuang L.Z., Zhang J.S. (2016). Correlations between stress corrosion cracking, grain boundary precipitates and Zn content of Al-Mg-Zn alloys’. J. Alloys Compd..

[19] Fang H.C., Chao H., Chen K.H. (2015). Effect of recrystallization on intergranular fracture and corrosion of Al-Zn-Mg-Cu-Zr alloy. J. Alloys Compd..

[20] Liu X.Y., Li M.J., Gao F., Liang S.X., Zhang X.L., Cui H.X. (2015). Effects of aging treatment on the intergranular corrosion behavior of Al-Cu-Mg-Ag alloy. J. Alloys Compd..

[21] Baydogan M., Cimenoglu H., Kayali E.S., Rasty J. (2008). Improved resistance to stress-corrosion-cracking failures via optimized retrogression and reaging of 7075-T6 aluminum sheets. Metall. Trans. A.

[22] Knight S.P., Birbilis N., Muddle B.C., Trueman A.R., Lynch S.P. (2010). Correlations between intergranular stress corrosion cracking, grain-boundary microchemistry, and grain-boundary electrochemistry for Al-Zn-Mg-Cu alloys. Corros. Sci..

[23] Oliveira A.F., de Barros M.C., Cardoso K.R., Travessa D.N. (2004). The effect of RRA on the strength and SCC resistance on AA7050 and AA7150 aluminium alloys. Mater. Sci. Eng. A.

[24] Chen J.F., Zhang X.F., Zou L.C., Yu Y., Li Q. (2016). Effect of precipitate state on the stress corrosion behavior of 7050 aluminum alloy. Mater. Charact..

[25] Peng G.S., Chen K.H., Chen S.Y., Fang H.C. (2013). Influence of dual-RRA temper on the exfoliation corrosion and electrochemical behavior of Al-Zn-Mg-Cu alloy. Mater. Corros..

[26] Buha J., Lumley R.N., Crosky A.G., Hono K. (2007). Secondary precipitation in an Al-Mg-Si-Cu alloy. Acta Mater..

[27] Buha J., Lumley R.N., Crosky A.G. (2008). Secondary ageing in an aluminium alloy 7050. Mater. Sci. Eng. A.

[28] Guyot P., Cottignies L. (1996). Precipitation kinetics, mechanical strength and electrical conductivity of AlZnMgCu alloys. Acta Mater..

[29] Li J.F., Jia Z.Q., Li C.X., Birbilis N., Cai C. (2009). Exfoliation corrosion of 7150 Al alloy with various tempers and its electrochemical impedance spectroscopy in EXCO solution. Mater. Corros..

[30] Polmear I.J. (2004). A century of age hardening. Mater. Forum.

[31] Nicolas M.N., Deschamps A. (2003). Characterisation and modelling of precipitate evolution in an Al-Zn-Mg alloy during non-isothermal heat treatments. Acta Mater..

[32] Hutchinson C.R., Gouné M.G., Redjaimia A. (2007). Selecting non-isothermal heat treatment schedules for precipitation hardening systems: An example of coupled process-property optimization. Acta Mater..

[33] Staley J.T. (2007). Precipitation Hardenable Alloy, e.g., Seven Thousand Series Aluminum Alloy, Aging Involves Heating Alloy at Continuous Rate of Increasing Temperatures Time to Age the Alloy. U.S. Patent.

[34] Jiang J.T., Tang Q.J., Yang L., Zhang K., Yuan S.J., Zhen L. (2016). Non-isothermal ageing of an Al-8Zn-2Mg-2Cu alloy for enhanced properties. J. Mater. Process. Technol..

[35] Jiang J.T., Xiao W.Q., Yang L., Shao W.Z., Yuan S.J., Zhen L. (2014). Ageing behavior and stress corrosion cracking resistance of a non-isothermally aged Al-Zn-Mg-Cu alloy. Mater. Sci. Eng. A.

[36] Li J.F., Zheng Z.Q., Li S.C., Chen W.J., Ren W.D., Zhao X.S. (2007). Simulation study on function mechanism of some precipitates in localized corrosion of Al alloys. Corros. Sci..

[37] Li H.Z., Yao S.C., Liang X.P., Chen Y.H., Liu C., Huang L. (2016). Grain boundary pre-precipitation and its contribution to enhancement of corrosion resistance of Al-Zn-Mg alloy. Trans. Nonferrous Met. Soc. China.

[38] Wang Z.T., Tian R.Z. (2000). Handbook of Aluminum Alloy and Its Working.

[39] Maitra S., English G. (1982). Environmental factors affecting localized corrosion of 7075-T7351 aluminum alloy plate. Metall. Trans. A.

[40] Chen S.Y., Chen K.H., Peng G.S., Jia L., Dong P.X. (2012). Effect of heat treatment on strength, exfoliation corrosion and electrochemical behavior of 7085 aluminum alloy. Mater. Des..

[41] Marlaud T., Deschamps A., Bley F., Lefebvre W., Baroux B. (2010). Influence of alloy composition and heat treatment on precipitate composition in Al-Zn-Mg-Cu alloys. Acta Mater..

[42] Kannan M.B., Raja V. (2007). Influence of heat treatment and scandium addition on the electrochemical polarization behavior of Al-Zn-Mg-Cu-Zr alloy. Metall. Trans. A.

[43] ASTM G34-01 (Reapproved 2013) (2013). Standard Test Method for Exfoliation Corrosion Susceptibility in 2XXX and 7XXX Series Aluminum Alloys (EXCO Test).

